# Pulsatilla Pratensis

**Published:** 1898-10

**Authors:** 


### HOMŒOPATHIC PHYSICIAN,

A MONTHLY JOURNAL OF

HOMŒOPATHIC MATERIA MEDICA AND CLINICAL MEDICINE.

"If our school ever gives up the strict inductive method of Hahnemann, we are lost, and deserve only to be mentioned as a caricature in the history of medicine."—CONSTANTINE HERING.

Vol. XVIII. OCTOBER, 1898. No. 10.

### EDITORIAL.

PULSATILLA PRATENSIS or Pulsatilla Nigricans is the greatest remedy in the materia medica of Homœopathy for the diseases and complaints of women. Its symptoms are characterized by rapid change from one to another, and the same symptom by rapid change from one part of the body to another. Variability of symptom is its characteristic. Dr. Henry N. Guernsey used to say of it that its variability was suggested by its character as a flower, being easily blown in different directions by the slightest current of air, owing to its slender stalk, and hence called the wind-flower. The keynotes given for it by Dr. Guernsey were many, and among the most striking indications of any that are to be found in our materia medica. These indications will be given as soon as the notes of Dr. Lippe upon it are finished.

On account of the striking character of its indications, it is more frequently called to mind in the troubles of women than any other remedy. It is, therefore, more frequently prescribed, though not always indicated when given, and consequently it is much abused. In this respect it calls to mind Aconite.

According to Dr. Lippe, the three great characteristics of Pulsatilla are peevishness, chilliness, and thirstlessness. It has one great characteristic running all through it. This is the tendency to weep. Many drugs have this tendency to weep, but Pulsatilla exceeds them all. Alumina has the same characteristic. The Pulsatilla patient is gloomy, melancholy, and full of cares. She constantly weeps.

Petroleum has sadness, despondency, and inclination to weep. Silicea, trifles irritate him very much. The Pulsatilla patient has anguish about the heart and desire for suicide. Aconite has anguish about the heart, with palpitation. The Pulsatilla patient has tremulous anguish, as if from approaching death. This is similar to Aconite.

Intellectual labor quickly fatigues the Pulsatilla patient. Calcarea-carbonica has utter impossibility of intellectual labor. Nux-vomica has aversion to mental labor, laziness.

The editor has made quite a collection of symptoms depending on intellectual labor. These were published under "Calcarea-carbonica," in this journal for July, 1897, pages 240 and 241, to which the reader is referred.

The Pulsatilla patient gets diarrhœa from fright. This is similar to Gelsemium and Opium.

The patient gets soreness in one or both temples, as if from subcutaneous ulceration. This is similar to Arnica.

The patient has twitching tearing in the temple on which she lies. This twitching tearing is characteristic of Pulsatilla. Pulsatilla has pain in the head, as if the brain were lacerated, soon after waking.

Lachesis has pain all over the head on waking in the morning.

The headache of Pulsatilla is ameliorated by walking slowly in the open air. This amelioration from walking slowly in the open air is characteristic of Pulsatilla. It is Dr. Guernsey's keynote. Under Rhus-tox., the longer the patient walks the better he feels.

The Pulsatilla patient takes cold from getting the head wet with perspiration.

Belladonna and Silicea, the patient takes told from uncovering the head.

Belladonna, the patient takes cold from having the hair cut.

Pulsatilla has swelling and redness of eyelids in rheumatic patients.

Pulsatilla has lachrymation in the wind. This is similar to Phos. and Sil.

Pulsatilla has inflammation of the eye, with secretion of thick greenish yellow mucus and agglutination of the eyelids at night. The eyes are sunken. Mercurius has violent inflammation of the eyes.

Fistula lachrymalis, with thick, heavy greenish yellow pus on pressing the tumor. In this connection the reader is referred to the case of fistula lachrymalis related under Silicea in the February number of this journal at page 50.

Calcarea, according to Dr. Lippe, is often indicated in fistula lachrymalis.

Pulsatilla has a sensation of a veil before the eyes, and the patient must continually wipe the eyes. This is a characteristic of Pulsatilla, and is one of Dr. Guernsey's keynotes.

The Pulsatilla patient has ulceration of the external wing of the nose, emitting a watery humor. This is a characteristic of Puls.

Pulsatilla has greenish yellow fœtid discharge from the nose.

Nux-vomica has stoppage of the nose during an attack of coryza.

Pulsatilla has stoppage of nose in warm room and free, open nostrils in the open air. Nux-vomica has coryza, with stoppage of the nose and dryness, in the warm room, and watery discharge in the open air.

All Pulsatilla discharges are thick and greenish yellow.

Pulsatilla has bleeding of the nose with suppressed menses.

Pulsatilla has alternate redness and paleness of the face. It generally has redness of the right cheek. It also has heat of the right hand.

Chamomilla has one red cheek and the other one pale.

Lachesis has one cheek red and the other pale.

Ferrum has red cheeks with paleness of rest of face.

Moschus has redness of right cheek without apparent heat, and paleness of left cheek with heat.

Bryonia has round red spot on one cheek.

